# Antibody titres elicited by the 2018 seasonal inactivated influenza vaccine decline by 3 months post‐vaccination but persist for at least 6 months

**DOI:** 10.1111/irv.13072

**Published:** 2022-11-30

**Authors:** Francesca L. Mordant, Olivia H. Price, Rajeev Rudraraju, Monica A. Slavin, Caroline Marshall, Leon J. Worth, Heidi Peck, Ian G. Barr, Sheena G. Sullivan, Kanta Subbarao

**Affiliations:** ^1^ Department of Microbiology and Immunology University of Melbourne at the Peter Doherty Institute for Infection and Immunity Melbourne Australia; ^2^ World Health Organization Collaborating Centre for Reference and Research on Influenza at the Peter Doherty Institute for Infection and Immunity Melbourne Australia; ^3^ Department of Infectious Diseases and Infection Prevention Peter MacCallum Cancer Centre Melbourne Australia; ^4^ National Centre for Infections in Cancer (NCIC), Sir Peter MacCallum Department of Oncology University of Melbourne Melbourne Australia; ^5^ Immunocompromised Host Infection Service Royal Melbourne Hospital Melbourne Australia; ^6^ Department of Infectious Diseases University of Melbourne at the Peter Doherty Institute for Infection and Immunity Melbourne Australia; ^7^ Infection Prevention and Surveillance Service Melbourne Health Melbourne Australia; ^8^ Victorian Healthcare Associated Infection Surveillance System (VICNISS) Royal Melbourne Hospital at the Peter Doherty Institute for Infection and Immunity Melbourne Australia

**Keywords:** antibody, influenza, kinetics, serosurvey, vaccine

## Abstract

**Background:**

In Australia, seasonal inactivated influenza vaccine is typically offered in April. However, the onset, peak and end of a typical influenza season vary, and optimal timing for vaccination remains unclear. Here, we investigated vaccine‐induced antibody response kinetics over 6 months in different age groups.

**Methods:**

We conducted a prospective serosurvey among 71 adults aged 18–50 years, 15 community‐dwelling (‘healthy’) and 16 aged‐care facility resident (‘frail’) older adults aged ≥65 years who received the 2018 southern hemisphere vaccines. Sera were collected at baseline, and 1, 2, 4, and 6 months post‐vaccination. Antibody titres were measured by haemagglutination inhibition or microneutralisation assays. Geometric mean titres were estimated using random effects regression modelling and superimposed on 2014–2018 influenza season epidemic curves.

**Results:**

Antibody titres peaked 1.2–1.3 months post‐vaccination for all viruses, declined by 3 months post‐vaccination but, notably, persisted above baseline after 6 months in all age groups by 1.3‐ to 1.5‐fold against A(H1N1)pdm09, 1.7‐ to 2‐fold against A(H3N2), 1.7‐ to 2.1‐fold against B/Yamagata and 1.8‐fold against B/Victoria. Antibody kinetics were similar among different age groups. Antibody responses were poor against cell‐culture grown compared to egg‐grown viruses.

**Conclusions:**

These results suggest subtype‐specific antibody‐mediated protection persists for at least 6 months, which corresponds to the duration of a typical influenza season.

## 1 INTRODUCTION

Vaccination is the cornerstone of the public health strategy for preventing severe influenza illness. Currently, inactivated influenza vaccine (IIV) is the only type of influenza vaccine licenced in Australia and is subsidised under the National Immunisation Program (NIP) for groups at risk of severe disease.^1^ Enhanced trivalent influenza vaccines, which were either high dose or adjuvanted, were added to the NIP in 2018 for older adults aged ≥65 years.

Annual influenza vaccination is recommended because the vaccine composition is updated each year to match circulating viruses and because antibody levels wane over time.^2^ In Australia, IIV is usually available in March, timed to precede the start of the influenza season in April/May. Seasons typically peak in August and end in October, but there may be substantial variability.^3^ For example, the 2017 influenza season started late but was associated with intense activity in primary care and hospitals, and widespread outbreaks in residential aged care facilities.^4^ By contrast, the 2018 influenza season was characterised by low activity but was followed by unusually high levels of interseasonal activity in 2018/2019 and an early and prolonged 2019 season.^5^ Such seasonal variation raises questions about optimal timing of influenza vaccination and whether vaccine‐induced immunity is likely to persist for the duration of the season.

A post‐vaccination geometric mean serum antibody titre ≥40, measured by haemagglutination inhibition (HI) assay, is the accepted correlate of protection for influenza vaccines, required by regulatory authorities for licencing.^6^ However, age,^7^ sex,^8^ body mass index (BMI),^9^ birth year^10^ and influenza vaccination history^11^ all influence the magnitude of the antibody response. Haemagglutinin (HA)‐ and neuraminidase (NA)‐specific antibody levels peak around 2–6 weeks post‐vaccination and decline to pre‐season levels thereafter.^12^, ^13^, ^14^ A systematic review and meta‐analysis of antibody responses in older adults found consistent evidence for decay in titres from 21–42 days to 1 year post‐vaccination.^2^ Another review found titres did not decline faster in older adults compared with younger adults aged ≤65 years.^15^ In most of the studies included in these analyses, titres were measured 1 and 6 months post‐vaccination but not between.^16^, ^17^, ^18^, ^19^, ^20^, ^21^, ^22^, ^23^, ^24^, ^25^ The decline between 1 and 6 months has not been well characterised. Where more frequent intervals have been examined, they have been limited to community‐dwelling ‘healthy’ older adults or ‘frail’ older adults without comparison of the two,^26^ have not compared older and younger adults^27^ or have not compared responses to quadrivalent (QIV) or enhanced trivalent vaccine in older adults.^28^, ^29^, ^30^

In this prospective serosurvey, we collected sera from adults aged ≤50 years and older adults aged ≥65 years at baseline and 1, 2, 4 and 6 months post‐vaccination to investigate the kinetics of antibody decay from 1 to 6 months. We superimposed our estimates on five influenza seasonal epidemic curves to investigate whether antibody levels persist for the duration of a typical influenza season. We also compared the kinetics between age groups, and between healthy and frail older adults. Additionally, vaccine antigens amplified in embryonated hens' eggs can acquire egg‐adaptive mutations in the HA; therefore, we compared antibody titres against the egg‐grown vaccine antigens with those against equivalent cell‐culture grown viruses that lack egg‐adaptive changes and are therefore more representative of clinical isolates.

## 2 MATERIALS AND METHODS

### 2.1 Study design

Ethics approval was granted by the Human Research Ethics Committee at the Royal Melbourne Hospital (RMH), Melbourne, Australia. Staff and volunteers at RMH and the Peter MacCallum Cancer Centre (PMCC) and residents of West Gippsland Healthcare Group aged‐care facilities (ACF) were recruited at each site's vaccination programme. Participants were classified as (1) adults aged 18–50 years; (2) community‐dwelling ‘healthy’ older adults aged ≥65 years or (3) ‘frail’ older adults aged ≥65 years. Eligibility was based on (i) being willing and able to provide informed consent; (ii) having no prior contraindications to the influenza vaccine; (iii) having no recent or current fever above 38°C; (iv) having no recent immunosuppressive treatment and (v) not having had already received the 2018 influenza vaccine. Age, sex, height, weight, medical history and vaccination history were collected at baseline; all data were self‐reported.

### 2.2 Vaccination

All participants received the 2018 southern hemisphere IIV appropriate for their age group and provided by their workplace or residence. Participants aged 18–50 years received standard dose QIV containing 15 μg of each HA (Afluria®Quad, Seqirus, Australia, or FluQuadri™, Sanofi, France). Participants aged ≥65 years received either Fluzone® High‐Dose (Sanofi) TIV containing 60 μg of each HA, or FLUAD® (Seqirus) adjuvanted TIV with 15 μg of each HA and formulated with MF59C.1. The vaccine compositions included A/Michigan/45/2015 (H1N1)pdm09‐like, A/Singapore/INFIMH‐16‐0019/2016 (H3N2)‐like and B/Phuket/3073/2013‐like viruses for TIV, and B/Brisbane/60/2008‐like virus included for QIV.

### 2.3 Sample collection

Baseline blood samples were collected just prior to vaccination. Follow‐up samples were collected at 1, 2, 4 and 6 months post‐vaccination. An additional sample was collected 12 months post‐vaccination from a subset of participants who could be contacted.

### 2.4 Serological assays

Sera were treated with 600 μl receptor‐destroying enzyme (RDE) (Denka‐Seiken) per 200 μl serum, adsorbed with 5% turkey red blood cells (tRBC), and stored at 4°C. Sera were diluted twofold from 1:5 to 1:5120 to measure antibody titres to A(H1N1)pdm09, B/Yamagata and B/Victoria strains by HI assay using tRBC; titres against A(H3N2) strains were measured by microneutralisation (MN) assay.^31^ Sera were tested against antigens that were included in the vaccines that the participants received. We compared antibody titres against the egg‐grown vaccine antigens with those against equivalent cell‐culture grown viruses that are considered more representative of clinical isolates. Circulating influenza A(H3N2) viruses have diversified into several genetic clades. The A(H3N2) vaccine component in 2018 was a clade 3C.2a1 virus (A/Singapore/INFIMH‐16‐0019/2016). To investigate the breadth of immunity elicited by the vaccine, we assessed the antibody response to a range of cell‐culture grown A(H3N2) viruses from different clades by MN assay and modelled the response using a linear mixed‐effects model. Cell‐culture grown A(H1N1)pdm09, B/Yamagata and B/Victoria viruses were propagated in Madin‐Darby Canine Kidney (MDCK) cells; A(H3N2) viruses were propagated in MDCK‐SIAT cells (MDCK cells expressing human 2,6‐sialtransferase). Influenza B viruses were ether split.^32^ HI titres were calculated as the reciprocal dilution of the last well with complete HI activity; MN titres were calculated as the reciprocal dilution of the last well where 50% of MDCK‐SIAT cells were infected. The lower limit of detection for HI titres was 5 and for MN titres was 10; a negative result was assigned a value of half the lower limit of detection for calculation purposes. Baseline, 1‐, 2‐, 4‐ and 6‐month samples were measured in the same assay; 12‐month samples were measured in an assay alongside baseline, 1‐ and 6‐month samples. Seroconversion was defined as a ≥fourfold rise in titre and seropositivity by titres ≥40.

### 2.5 Statistical analysis

All data cleaning, modelling and visualisation were completed in R version 3.5.1 (R Foundation for Statistical Computing, Vienna, Austria). Differences in baseline characteristics between groups were assessed using the Chi‐square test for categorical outcomes and one‐way ANOVA for continuous outcomes. The significance level was set at 0.05. Seropositivity was defined as a HI geometric mean titre (GMT) of at least 40; seroconversion was defined as a minimum four‐fold rise in post‐vaccination HI antibody titre. Average antibody decay was modelled as time since vaccination on log_2_ HI titre using a linear mixed‐effect model.^33^ Using Akaike Information Criterion and prior assumptions about the antibody kinetic trajectory, the model included a natural cubic spline with three knots placed at quartiles. To compare decay curves between groups, an interaction term was included between time and group. Additional covariates (age and sex) were added to the model and their goodness of fit tested by likelihood ratio test. Predicted GMTs and 95% prediction intervals (95%PI) were estimated using a semi‐parametric bootstrap for mixed models.^33^ Differences in antibody decay curves were estimated across groups by type III ANOVA (using Satterthwaite's degrees of freedom method^34^). Older adult participants received e‐TIV that did not contain a B/Victoria component, so their antibody titres measured against B/Victoria were not included in analyses. Log_2_ HI titres were back‐transformed for ease of interpretation.

Epidemic curves were produced using National Notifiable Diseases Surveillance System (NNDSS) notification data from the state of Victoria. These were smoothed using a 3‐week moving average and superimposed on predicted antibody titre curves.

## 3 RESULTS

We recruited 71 adults aged 18–50 years, 15 healthy and 16 frail older adults aged ≥65 years, representing 44% recruitment uptake (Figure S1). The overall completion rate was 91% (93/102 participants). The median age of participants was 30 (range: 20–49) years in the young adult group, 72 (65–86) years in the healthy older adult group and 84.5 (68–94) years in the frail older adult group. A majority (89.2%) of participants reported having received at least one dose of IIV within the last 5 years; four reported no influenza vaccination in the last 5 years; seven could not recall. Most participants had a BMI ≤ 30 and were not considered obese (92.2%; *n* = 94). Due to the high rate of prior vaccination and low obesity, the effects of these variables on the antibody response were not explored.

### 3.1 Antibody titres against vaccine antigens waned over time but persisted for at least 6 months

**FIGURE 1:**
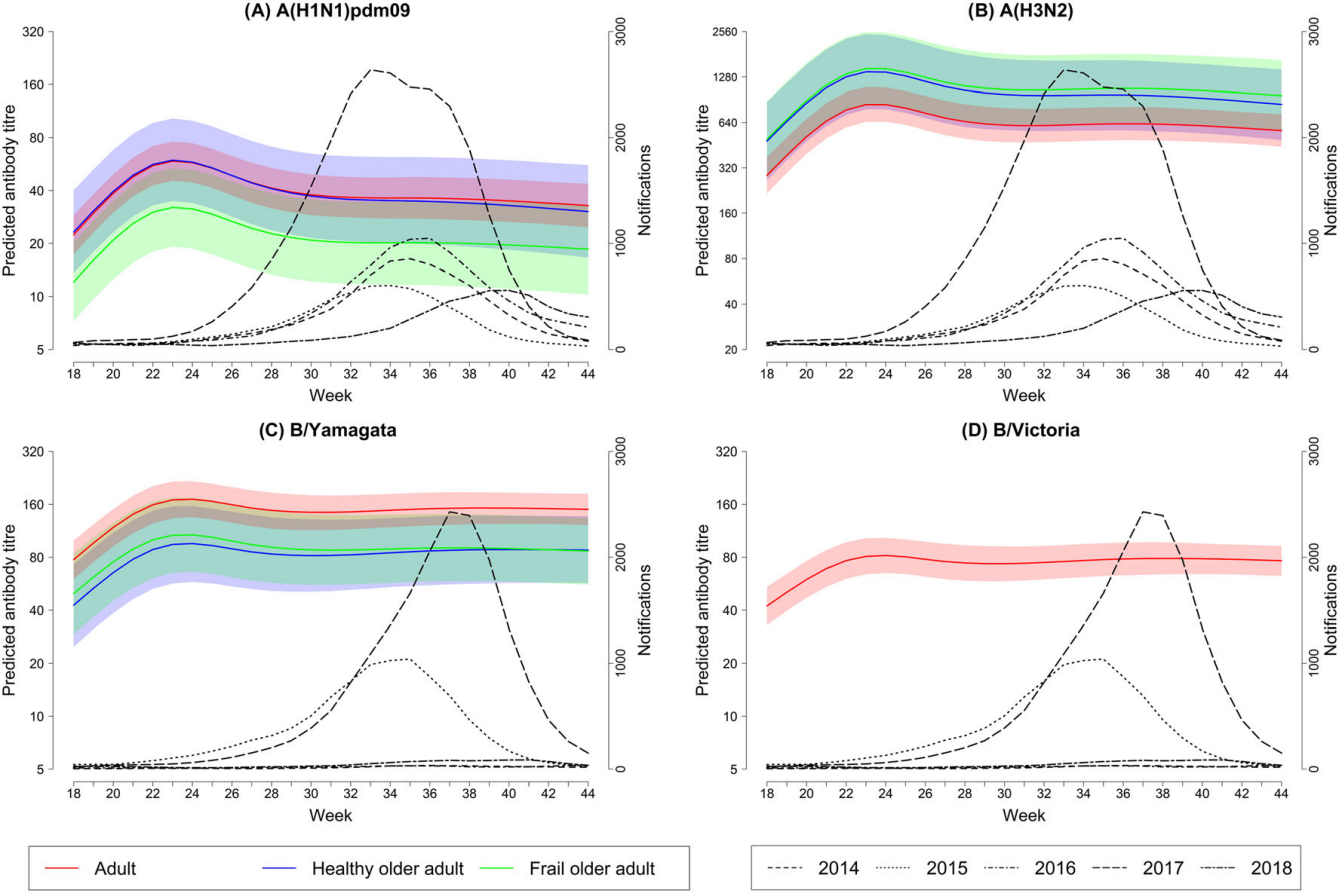
Concordance between influenza season timing and predicted antibody decay. Sera were collected from participants in different age groups at baseline, and 1, 2, 4 and 6 months post‐vaccination time‐points; serum antibody titres to A(H1N1)pdm09, B/Yamagata and B/Victoria were measured by haemagglutination inhibition assay, and titres to A(H3N2) were measured by microneutralisation assay. Predicted geometric mean titres (GMT) with 95% prediction intervals (95%PI) against (A) A(H1N1)pdm09, (B) A(H3N2), (C) B/Yamagata and (D) B/Victoria vaccine antigens, by age group, modelled using a linear mixed‐effect model. National Notifiable Diseases Surveillance System (NNDSS) case notification data from the state of Victoria, Australia, were used to generate epidemic curves from 2014–2018 influenza seasons, which were superimposed onto antibody kinetics curves.

Predicted GMTs of influenza A and B‐specific antibodies, fold‐rises in titre from baseline, and rates of seroconversion are presented in Tables 1 and 2. Antibody titre decay curves were superimposed onto influenza case notification data from 2014–2018 seasons (Figure 1). Peak predicted GMTs against egg‐grown antigens were observed between 1.2 and 1.3 months post‐vaccination in all groups against all vaccine antigens. This occurred before the peak in any influenza season, which fell between week 32–34 for influenza A and week 36–38 for influenza B. Titres declined by 3 months post‐vaccination but remained steady thereafter. Titres remained above baseline by 1.3‐ to 1.5‐fold against A(H1N1)pdm09, 1.7‐ to 2‐fold against A(H3N2), 1.7‐ to 2.1‐fold against B/Yamagata in all groups and 1.8‐fold against B/Victoria in the adult group 6 months after vaccination, corresponding to the duration of the 2014–2018 seasons. The 2018 season was unusually prolonged, with case notifications still increasing by week 44 (Figure 1). An additional serum sample was collected from a subset of participants 12 months post‐vaccination; 37 adults aged 18–50 years (52% of original cohort), 11 healthy older adults (73%) and 11 frail older adults aged ≥65 years (69%). Titres had returned to baseline for A(H1N1)pdm09 and A(H3N2) but remained above baseline for B/Yamagata and B/Victoria egg‐grown viruses (data not shown).

**TABLE 1:** Antibody titres against influenza A vaccine antigens and equivalent cell‐culture grown viruses in adults, healthy older adults and frail older adults aged ≥65 years at baseline and 1, 2, 4 and 6 months post‐vaccination

Group	Timepoint (months p.v.)	A(H1N1)pdm09 egg			A(H1N1)pdm09 cell			A(H3N2) egg			A(H3N2) cell		
		GMT (95%PI)	FD^a^	SC^b^	GMT (95%PI)	FD^a^	SC^b^	GMT (95%PI)	FD^a^	SC^b^	GMT (95%PI)	FD^a^	SC^b^
Adults 18–50 years	Baseline	22.4 (17.4, 28.7)	‐	‐	28.4 (22.2, 36.2)	‐	‐	285.1 (214.2,376.3)	‐	‐	13.7 (11.2, 16.7)	‐	‐
	1	57.1 (44.3, 73.4)	2.6	25.7	74.7 (58.3, 95.2)	2.6	34.3	805.2 (608.9,1062.4)	2.8	37.1	15.4 (12.8, 18.6)	1.1	4.3
	2	45.5 (35.1, 58.9)	2	21.4	64 (49.7, 81.6)	2.2	22.9	698.8 (534,911.1)	2.5	30.0	14.9 (12.4, 17.8)	1.1	2.9
	4	36.2 (27.7, 47.4)	1.6	15.9	53.1 (40.9, 68.5)	1.9	18.8	626.5 (485.5,804.5)	2.2	23.2	13.1 (11, 15.7)	1	2.9
	6	32.8 (24.8, 43.3)	1.5	10.4	47.6 (36.3,62.5)	1.7	13.4	563.8 (440,725.1)	2	23.9	13.3 (11.2, 15.8)	1	1.5
Healthy older adults ≥65 years	Baseline	23.2 (13.7, 39.9)	‐	‐	18 (10.7, 30.2)	‐	‐	480.4 (263.4,885.9)	‐	‐	18.8 (12.4, 28.5)	‐	‐
	1	58 (34.1, 99.4)	2.5	20.0	45.4 (27, 76.9)	2.5	40.0	1,329.2 (745.3,2374.4)	2.8	20.0	20.4 (13.8, 30.5)	1.1	0.0
	2	45.3 (26.5, 78.9)	2	0.0	37.3 (21.9, 63)	2.1	20.0	1,130.1 (646.3,1970.1)	2.4	20.0	18.9 (12.9, 27.9)	1	0.0
	4	34.8 (19.9, 61.7)	1.5	0.0	28.5 (16.5, 49.7)	1.6	7.1	972.6 (570.9,1657.3)	2	14.3	15.4 (10.6, 22.3)	0.8	0.0
	6	30.3 (16.7, 55.8)	1.3	0.0	23.5 (13.1, 42.6)	1.3	0.0	840 (487.4,1442.6)	1.7	14.3	14.4 (9.9, 20.9)	0.8	0.0
Frail older adults ≥65 years	Baseline	12.1 (7.3, 20.3)	‐	‐	14.3 (8.6, 23.5)	‐	‐	494.2 (275.4,880.9)	‐	‐	12.6 (8.4, 18.9)	‐	‐
	1	31.1 (18.6, 52.4)	2.6	53.3	36.2 (21.8, 59.9)	2.5	26.7	1,391.5 (796.2,2450.1)	2.8	60.0	14.3 (9.7, 21.1)	1.1	13.3
	2	25 (14.9, 42.6)	2.1	46.7	30 (17.9, 49.5)	2.1	20.0	1,204 (702.5,2070.1)	2.4	60.0	13.9 (9.5, 20.2)	1.1	13.3
	4	20.2 (11.6, 35.6)	1.7	14.3	23.2 (13.5, 39.4)	1.6	7.1	1,073.2 (634.4,1819)	2.2	42.9	12.4 (8.6, 17.8)	1	0.0
	6	18.5 (10.2, 34.1)	1.5	16.7	19.4 (10.9, 34.6)	1.4	8.3	960 (561.5,1651.2)	1.9	8.3	12.7 (8.8, 18.2)	1	0.0

^a^ Mean fold difference in predicted GMT at each time‐point relative to predicted GMT at baseline.

^b^ Seroconversion; the % of participants with a ≥fourfold rise in titre from baseline.

**TABLE 2:** Antibody titres against influenza B vaccine antigens and the equivalent cell‐culture grown viruses in adults, healthy older adults and frail older adults aged ≥65 years at baseline and 1, 2, 4 and 6 months post‐vaccination

Group	Timepoint (months p.v.)	B/Yamagata egg			B/Yamagata cell			B/Victoria egg			B/Victoria cell		
		GMT (95%PI)	FD^a^	SC^b^	GMT (95%PI)	FD^a^	SC^b^	GMT (95%PI)	FD^a^	SC^b^	GMT (95%PI)	FD^a^	SC^b^
Adults 18–50 years	Baseline	77.4 (60.1, 99.9)	‐	‐	26.3 (20.5, 33.4)	‐	‐	42.2 (32.9, 54.3)	‐	‐	12.7 (10.3, 15.7)	‐	‐
	1	163.9 (128.3,208.5)	2.1	20.0	49.7 (39.2, 62.8)	1.9	17.1	78.4 (61.9, 99.5)	1.9	25.7	22.9 (18.8, 27.9)	1.8	24.3
	2	154.1 (121.7,194.8)	2	17.1	48.3 (38.5, 60.9)	1.8	17.1	76.1 (60.8, 95.5)	1.8	22.9	20 (16.5, 24.1)	1.6	20.0
	4	150.2 (120.7,186.9)	1.9	23.2	45.6 (36.4, 56.9)	1.7	14.5	77.8 (62.8, 96.3)	1.8	21.7	23.2 (19.5, 27.6)	1.8	23.2
	6	149.7 (121.8,183.5)	1.9	20.0	43.8 (35.2, 54.4)	1.7	13.8	76.3 (62.7, 93)	1.8	20.9	24.9 (21.2, 29.1)	2	29.9
Healthy older adults ≥65 years	Baseline	42.6 (24.8, 72.8)	‐	‐	18.1 (10.8, 30.4)	‐	‐	‐	‐	‐	‐	‐	‐
	1	91.2 (54.5,151.7)	2.1	26.7	34.5 (21, 56.8)	1.9	20.0	‐	‐	‐	‐	‐	‐
	2	86.7 (53.2,141.5)	2	20.0	33.8 (20.8, 55)	1.9	0.0	‐	‐	‐	‐	‐	‐
	4	86.3 (54.7,136.6)	2	15.4	32.4 (20.2, 51.8)	1.8	0.0	‐	‐	‐	‐	‐	‐
	6	87.8 (56.7,137)	2.1	25.0	31.7 (19.6, 50.4)	1.7	0.0	‐	‐	‐	‐	‐	‐
Frail older adults ≥65 years	Baseline	49.7 (29.4, 84.7)	‐	‐	20.2 (12.2, 33.2)	‐	‐	‐	‐	‐	‐	‐	‐
	1	103.3 (62.7,170.9)	2.1	40.0	38.1 (23.4, 61.8)	1.9	20.0	‐	‐	‐	‐	‐	‐
	2	95.5 (59.7,155.2)	1.9	20.0	36.9 (22.9, 58.4)	1.8	13.3	‐	‐	‐	‐	‐	‐
	4	90 (58.2,140.9)	1.8	23.1	34.7 (21.8, 54.4)	1.7	23.1	‐	‐	‐	‐	‐	‐
	6	86.6 (56.2,134.3)	1.7	10.0	33.2 (20.6, 52.2)	1.6	10.0	‐	‐	‐	‐	‐	‐

^a^ Mean fold difference in predicted GMT at each time‐point relative to predicted GMT at baseline.

^b^ Seroconversion; the % of participants with a ≥fourfold rise in titre from baseline.

### 3.2 Post‐vaccination antibody titres against influenza vaccine antigens and the equivalent cell‐culture grown viruses by age group

**FIGURE 2:**
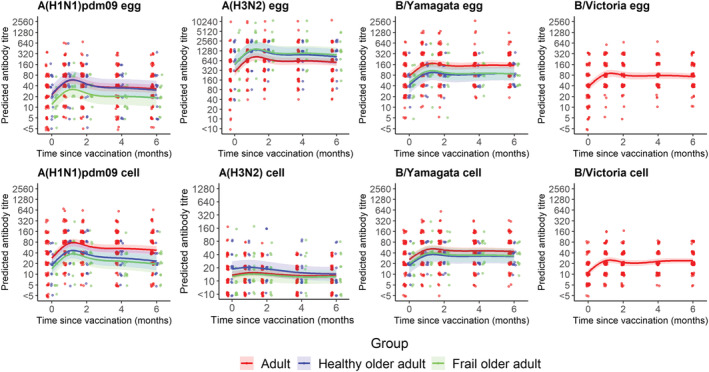
Serum antibody responses to egg and cell‐culture grown antigens over 6‐months post‐vaccination. Sera were collected from participants in different age groups at baseline, and 1, 2, 4 and 6 months post‐vaccination time‐points. Antibody titres were measured against the four vaccine antigens (‘egg’; top row) and cell‐grown equivalent strains (bottom row); titres against A(H1N1)pdm09, B/Yamagata and B/Victoria were measured by haemagglutination inhibition assay and against A(H3N2) by microneutralisation assay. Lines show the predicted geometric mean titres (GMT); shaded areas show 95% prediction intervals (95%PI); points represent individual titre values. Points are jittered.

Antibody titres were measured against each antigen contained in the influenza vaccine and against the equivalent cell‐culture grown viruses (Figure 2).

In all age groups, predicted GMTs were higher against the egg‐grown viruses than against the equivalent cell‐culture grown viruses (predicted GMTs [95% PI] at 1‐month post‐vaccination: 944.0 [741.4–1195.4] vs. 15.9 [13.6–18.6] for A(H3N2); 139.0 [113.2–172.6] vs. 45.2 [37.2–55.2] for B/Yamagata; 78.4 [61.9–99.5] vs. 22.9 [18.8–27.9] for B/Victoria in the adult group only). Conversely, titres against A(H1N1)pdm09 were moderately lower against the egg‐grown viruses in the adult (57.1 [44.3–73.4] vs. 74.7 [58.3–95.2]) and frail older adult (31.1 [18.6–52.4] vs. 36.2 [21.8–59.9]) groups but not in the healthy older adult group (58.0 [34.1–99.4] vs. 45.4 [27.0–76.9]) 1‐month post‐vaccination.

The frail older adults consistently displayed the highest rates of seroconversion (≥fourfold rise in titre from baseline to 1‐month post‐vaccination) against A(H1N1)pdm09 (53.3%), A(H3N2) (60.0%) and B/Yamagata (40.0%) vaccine antigens.

Peak titres against all egg‐ and cell‐culture grown viruses were consistently observed 1‐month post‐vaccination in all age groups, except against cell‐culture grown B/Victoria in the adult group, which marginally increased from 22.9 [18.8–27.9] 1‐month post‐vaccination to 24.9 [21.2–29.1] 6‐months post‐vaccination (Figure 2).

Increases in antibody titres measured by fold‐rise from baseline to 1‐month post‐vaccination were similar among groups between egg‐ and cell‐culture grown A(H1N1)pdm09 (2.5‐ to 2.6‐fold), B/Yamagata (1.9‐ to 2.1‐fold) and B/Victoria (2.5–2.6) viruses; however, there was a greater difference in fold‐rise against egg‐grown and cell‐culture grown A(H3N2) viruses (2.8‐fold vs. 1.1‐fold, respectively). The proportion of participants who seroconverted against cell‐culture grown A(H3N2) was markedly lower than against egg‐grown viruses (4.3% vs. 37.1% in adults; 0% vs. 20% in healthy older adults; 13.3% vs. 60% in frail older adults).

The rate of antibody decay over time only differed between groups against cell‐culture grown A(H1N1)pdm09 virus (*p* = 0.028) where antibody levels in the older adult groups declined more rapidly than in the adults; titres dropped by 1.5‐fold from the peak after 4.8 months post‐vaccination in the adults, but after 2.4‐ and 2.8‐months post‐vaccination in the healthy and frail older adult groups, respectively.

### 3.3 Antibody responses against distinct A(H3N2) clade viruses

**FIGURE 3:**
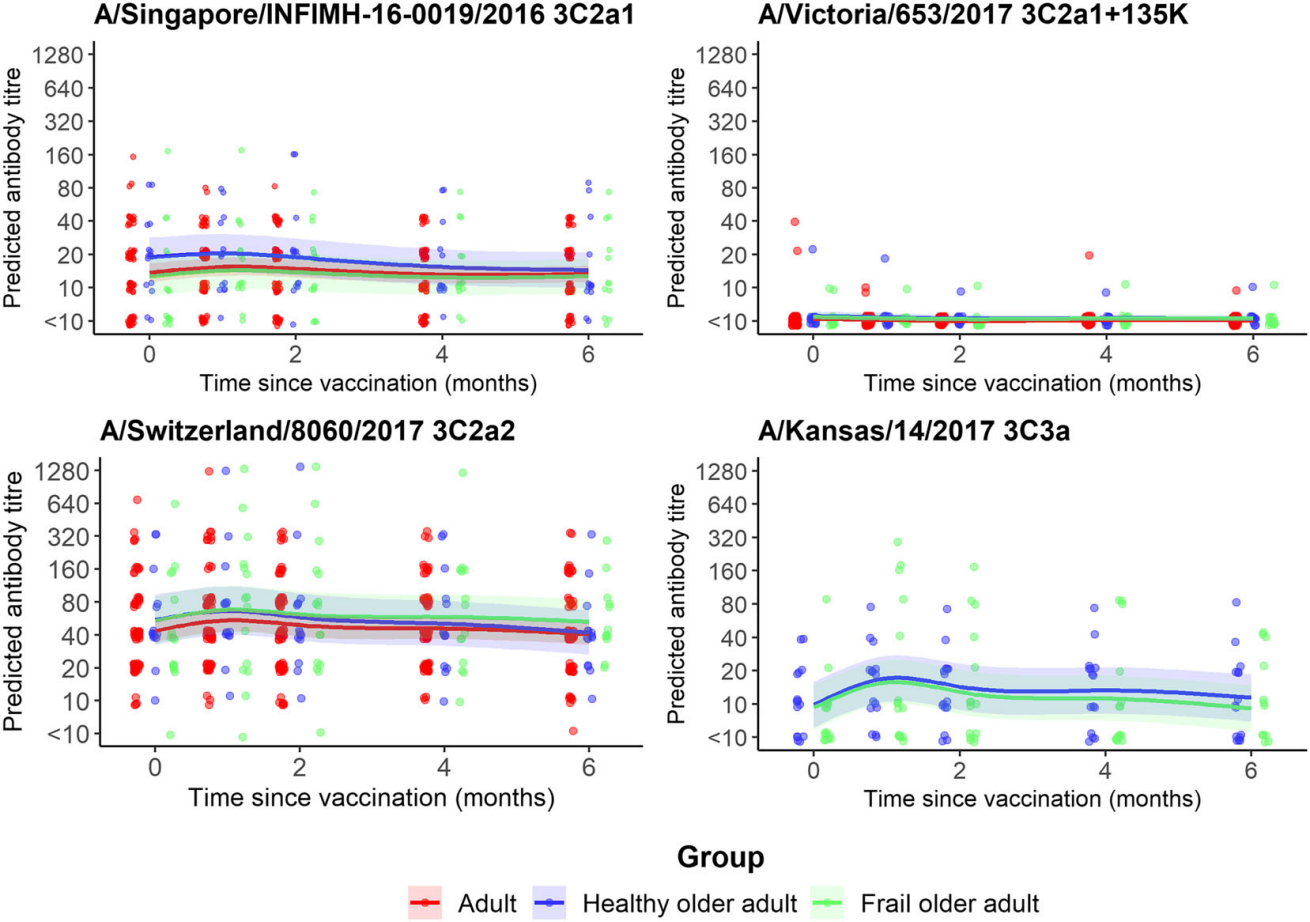
Differences in antibody response to 4 H3N2 reference antigens. Sera were collected from participants in different age groups at baseline and 1, 2, 4 and 6 months post‐vaccination. Serum antibody titres were measured by microneutralisation assay against cell‐culture grown A(H3N2) strains in four different A(H3N2) clades. Lines show the predicted geometric mean titres (GMT); shaded areas show 95% prediction intervals (95%PI); points represent individual titre values. Points are jittered.

Titres against other cell‐culture grown A(H3N2) viruses from distinct clades to the vaccine antigen (A/Singapore/INFIMH‐16‐0019/2016; clade 3C.2a1) were assessed (Figure 3).

Titres to the cell‐culture grown clade 3C.2a2 virus (A/Switzerland/8060/2017) were greater than those to the cell‐culture grown equivalent of the clade 3C.2a1 vaccine antigen, and there were modest increases in titre from baseline to 1‐month post‐vaccination of 1.2‐fold in adults and healthy older adults and 1.3‐fold in the frail older adults. The proportion of individuals who seroconverted was also greatest in the frail older adults (20%) compared to the healthy older adults (6.7%) and adults (4.3%). There was no response detected to the 3C.2a1b + 135K virus (A/Victoria/653/2017) with no rise in titre from baseline and no seroconverters in any age group. Titres against the 3C.3a virus (A/Kansas/14/2017) showed the greatest increase from baseline to 1‐month post‐vaccination, with a 1.7‐fold rise in both the healthy older adults (14.3% seroconversion) and frail older adults (6.3% seroconversion). Interestingly, 8.3% of older adults showed a ≥fourfold rise in titre from baseline to 6 months post‐vaccination, which was greater than 1‐month post‐vaccination.

### 3.4 Antibody response by sex

Among the adult group, antibody titres did not significantly differ by sex, except against the B/Yamagata vaccine antigen where titres were higher in males than females (*p* = 0.02), though the rate of antibody decay against this antigen did not differ (*p* = 0.72) (data not shown).

## 4 DISCUSSION

Overall, this study shows that the influenza vaccine‐induced antibody response declined after 3 months but importantly persisted above baseline at 6 months post‐vaccination, corresponding to the duration of a typical influenza season. The antibody response varied by antigen; however, we generally did not observe any difference between age groups or by sex.

Notably, our study fills a gap in the literature by defining the timeframe wherein antibody titres begin to decline; earlier studies could only infer that the decline occurs between 1 and 6 months post‐vaccination.^16^, ^17^, ^18^, ^19^, ^20^, ^21^, ^22^, ^23^, ^24^, ^25^ We also compared titres between healthy young adults and healthy and frail older adults using licenced QIV and eTIV in Australia.^28^, ^29^, ^30^

Our finding that antibody titres persisted above baseline for at least 6 months, and even up to 12 months post‐vaccination against influenza B antigens, is consistent with a previous study that showed anti‐influenza HA and NA antibody titres in younger adults remained high after 18 months and may even persist over multiple seasons.^35^ A study in older adults observed sustained titres over a 12‐month period following IIV,^25^ although this contradicted other observations of unreliable year‐round persistence of antibody titres in older adults.^2^ Persistence of titres for at least 6 months following vaccination corresponded to the duration of the 2014–2017 influenza seasons in Victoria, Australia, but not 2018, which was unusually early and long.^36^ The decline of antibody titres against influenza A antigens between 6 and 12 months post‐vaccination, though titres against the influenza B antigens persisted for 12 months, may have implications for protection against influenza A viruses during late or prolonged influenza seasons.

We observed that the kinetics of antibody decay did not differ between adults aged 18–50 and healthy and frail older adults aged ≥65. This is consistent with a meta‐analysis that suggested antibody levels following influenza vaccination in older adults do not necessarily decline at a higher rate than younger adults as is commonly believed.^15^ Unfortunately, we could not perform a direct comparison of the antibody response between participant groups in this study after receiving a standard dose of QIV due to the introduction of eTIV for persons aged ≥65 in Australia for the 2018 season. A previous study comparing antibody responses between standard‐dose and high‐dose IIV recipients in a frail older adult population showed that the high‐dose vaccine elicited greater antibody titres after 1 month; however, there was little difference in the rate of antibody decline between 1 and 6 months.^24^ While we cannot make a clear comparison in our cohort, there may be minimal difference between the kinetics of the antibody response following standard‐dose and high‐dose IIV in older adults.

We observed that rates of seroconversion against influenza vaccine antigens were consistently greatest in the frail older adult group. Nunez et al.^37^ also observed higher seroconversion rates to influenza A in an older age group compared to a younger age group, and they noted that this was dependent on seropositivity status where HI titres <40 are considered ‘seronegative’ prior to vaccination. Individuals were not more or less likely to seroconvert based on age, but younger individuals were more likely to be seropositive prior to vaccination and therefore remain seropositive, without seroconverting.^37^

Antibody responses to the cell‐culture grown equivalent strains for all subtypes were equal to or lower than against the vaccine antigens in all age groups tested. The greatest difference in titres was observed between cell‐culture and egg‐grown A(H3N2) strains, and far fewer people seroconverted against the cell‐grown A(H3N2) virus than against the vaccine antigen. Furthermore, we observed consistently poor responses to cell‐grown A(H3N2) strains from antigenically distinct clades. These findings are consistent with a report of altered antibody titres between A(H3N2) vaccine strains and circulating viruses,^38^ which also noted that titres against circulating but not egg‐adapted vaccine strains were correlated with protection against infection. This effect is expected because propagation of the vaccine viruses during manufacturing can lead to major antigenic changes in the A(H3N2) HA protein as the virus adapts to growth in eggs, including a loss of glycosylation site from a T160K reversion or a L194P change in antigenic site B.^39^, ^40^, ^41^ These changes in antigenicity can result in the antibody response elicited by vaccination being targeted towards egg‐adapted A(H3N2) HA, and consequently being less cross‐reactive with circulating A(H3N2) viruses.^40^, ^42^ This is one of the causes of sub‐optimal vaccine effectiveness against A(H3N2) in recent years.^43^, ^44^, ^45^ Poor vaccine effectiveness against A(H3N2) has also been attributed to mismatch between vaccine and circulating strains.^46^, ^47^ Influenza viruses continuously evolve leading to substantial diversification of circulating A(H3N2) viruses^48^ with several antigenically distinct groups co‐circulating, making it difficult to select vaccine candidates that afford broad coverage.

While previous studies have suggested that the antibody response tends to be higher in females than in males,^49^ we observed slightly higher antibody responses in males to B/Yamagata only; however, our study consisted of a relatively small sample size therefore this result should be interpreted with caution. There were other limitations to this study. First, our cohort size was insufficiently powered to investigate other factors that could affect the dynamics of the antibody response following vaccination. We could not assess the effect of prior vaccination history because there were few vaccine‐naïve participants in our study. Previous studies have shown that titres are reduced or decrease more rapidly among highly vaccinated individuals.^11^, ^50^ We did not monitor participants for influenza infection during the study which may interfere with antibody kinetics. We also were unable to examine the effects of obesity that has been associated with higher initial rises, but steeper declines in titre.^9^ Finally, we were unable to compare responses to high dose or adjuvanted eTIVs in the healthy and frail older adults due to insufficient numbers and the observational nature of this study.

While the optimal timing for influenza vaccination remains uncertain, we found that antibody responses following influenza vaccination wane over time but, crucially, persist for the duration of a typical influenza season. Further research is required before changes to current policy regarding the timing for influenza vaccine rollout should be recommended. The consequences of delaying vaccination on overall vaccine uptake, immunological factors, logistical challenges of condensed vaccine rollout while achieving the same level of coverage, changes to the manufacturing process and inter‐season variability must all first be carefully considered. Our findings, taken together with the wide range of uncertainties regarding the optimal timing for influenza vaccination, suggest any policy changes at this stage may be premature.

## AUTHOR CONTRIBUTIONS

**Francesca L. Mordant:** Conceptualization; investigation; methodology; project administration; resources; validation; visualization; writing‐original draft; writing‐review and editing. **Olivia H. Price:** Data curation; formal analysis; methodology; resources; software; validation; visualization; writing‐original draft; writing‐review and editing. **Rajeev Rudraraju:** Conceptualization; investigation; methodology; project administration; resources; writing‐review and editing. **Monica A. Slavin:** Resources; writing‐review and editing. **Caroline Marshall:** Resources; writing‐review and editing. **Leon J. Worth:** Resources; writing‐review and editing. **Heidi Peck:** Resources; writing‐review and editing. **Ian G. Barr:** Conceptualization; methodology; resources; writing‐review and editing. **Sheena G. Sullivan:** Conceptualization; data curation; formal analysis; methodology; resources; software; visualization; writing‐review and editing. **Kanta Subbarao:** Conceptualization; funding acquisition; methodology; project administration; resources; supervision; writing‐original draft; writing‐review and editing.

## CONFLICT OF INTEREST

FM, CM and IB have shares in companies that produce influenza vaccines. OP, RR, MS, LW, HP, SS and KS report no potential conflict of interest.

### PEER REVIEW

The peer review history for this article is available at https://publons.com/publon/10.1111/irv.13072.

## Supporting information


**Figure S1:** CONSORT diagram

## Data Availability

The data that support the findings of this study are available from the corresponding author upon reasonable request.
