# Supplementary material for: Antibody titres elicited by the 2018 seasonal inactivated influenza vaccine decline by 3 months post‐vaccination but persist for at least 6 months
Source: Influenza Other Respir Viruses. 2022 Nov 30;17(1):e13072. doi: 10.1111/irv.13072 (PMC9835415; doi:10.1111/irv.13072)
Supplement: Supplementary file 1 — Figure S1: CONSORT diagram [file IRV-17-e13072-s001.docx]

## Supplementary Figure 1: CONSORT diagram
